# Influence of Hypnosis and Acupuncture on Perioperative Complications

**DOI:** 10.3390/healthcare13222992

**Published:** 2025-11-20

**Authors:** Jasmina Markovič-Božič, Meta Gradišar, Mihela Petovar, Polona Mušič, Nina Pirc, Joseph Meyerson, Maks Tušak, Andrej Lapoša, Matej Tušak, Alenka Spindler-Vesel

**Affiliations:** 1 Department of Anaesthesiology and Surgical Intensive Therapy, University Medical Centre Ljubljana, 1525 Ljubljana, Slovenia; meta.gradisar@kclj.si (M.G.); Mihaela.petovar@kclj.si (M.P.); polona.music@kclj.si (P.M.); nina.pirc@kclj.si (N.P.); alenka.spindler@guest.arnes.si (A.S.-V.); 2 Department of Anaesthesiology and Reanimation, Faculty of Medicine, University of Ljubljana, 1000 Ljubljana, Slovenia; 3 Medical Psychology Department, The Max Stern Yezreeal Valley College, Tel Aviv, Emek Yezreel, Tel Aviv 19300, Israel; hypnoclinic10@gmail.com; 4 Emergency Department, University Medical Centre Ljubljana, 1000 Ljubljana, Slovenia; maks.tusak@gmail.com; 5 Department of Plastic, Reconstructive, Aesthetic Surgery and Burns, University Medical Centre Ljubljana, 1525 Ljubljana, Slovenia; andrej.laposa@kclj.si; 6 Department of Surgery, Faculty of Medicine, University of Ljubljana, 1000 Ljubljana, Slovenia; 7 Faculty of Sport, University of Ljubljana, 1000 Ljubljana, Slovenia; tusakm@gmail.com

**Keywords:** spinal surgery, anaesthesia, antiemetic, acupuncture, hypnosis

## Abstract

**Background**: This randomised, single-centre study and original research manuscript aimed to evaluate whether perioperative hypnosis and acupuncture can reduce postoperative nausea and vomiting (PONV), opioid use, and other complications in spinal surgery compared to standard pharmacological management. **Methods**: In total, 60 patients undergoing spinal surgery were divided into three groups regarding antiemetic prevention: Hypnosis and acupuncture (HG), hypnosis, acupuncture, and antiemetic (HAG), and standard control with antiemetic (CG). Hypnosis was performed one day before surgery, or patients received premedication with midazolam on the day of surgery. Anaesthesia was induced and maintained with propofol and remifentanil. Acupuncture was performed bilaterally at points LI4 and PC6 after induction of anaesthesia. Postoperatively, the consumption of opioids and antiemetics, satisfaction and well-being, length of stay and complications were recorded. **Results**: In all groups, additional opioids were administered in the first hour after surgery (*p* = 0.4). In the ICU, only one patient in the HAG and two patients in the CG and HG required additional analgesics (*p* = 0.8). Overall satisfaction (9/9/0 vs. 10/6/4 vs. 9/7/3; *p* = 0.4) and well-being scores (10/8/0 vs. 13/5/2 vs. 13/5/1; *p* = 0.5) were high across all groups, with no significant differences. Two patients in CG experienced mild complications. The length of hospitalisation was similar (3 days in CG vs. 4 days in HAG and HG (*p* = 0.7). Only one patient in the HG required antiemetics; none were needed in CG or HAG (*p* = 0.4). **Conclusions**: Within the constraints of this exploratory single-centre trial, hypnosis appeared to provide anxiolytic benefits comparable to benzodiazepines, and intraoperative acupuncture did not increase PONV despite reduced pharmacological prophylaxis. No significant differences were detected in opioid and antiemetic consumption. Larger, adequately powered studies are needed to confirm these findings and optimise the timing and modality of non-pharmacological interventions.

## 1. Introduction

Perioperative complications are still part of everyday clinical practice despite major advances in medicine and great efforts to improve patient safety. In addition to pharmacological treatment, they can also be alleviated by non-pharmacological methods such as medical hypnosis and acupuncture. Acupuncture can be used in perioperative anaesthesia as acupuncture-assisted anaesthesia. It cannot provide adequate anaesthesia, but it can reduce the need for drugs from the anaesthetic triangle, namely volatile, intravenous anaesthetics and opioids [1,2]. Its use as part of a multimodal approach is of particular interest as it reduces the consumption of perioperative drugs and their side effects. This means lower overall costs for surgery and postoperative care [1,2]. Acupuncture helps to reduce complications due to inadequate postoperative analgesia. It has been shown to reduce pain after craniotomy, caesarean section, lumbar spine surgery and knee or hip arthroplasty [2,3]. Acupuncture stimulation of PC6 has similar efficacy in the prevention of PONV as standard antiemetics [4]. The acupuncture points LI4 and ST36 can also be used. Several studies have shown that the stimulation of one acupuncture point is less effective than the simultaneous stimulation of several acupuncture points [4]. The effect is independent of the time of application and can therefore be used before or after induction of anaesthesia [2,4,5].

Medical hypnosis is defined as a state of consciousness with focused attention and reduced peripheral awareness, characterised by an increased ability to respond to suggestion [6]. It is a non-pharmacological method that reduces perioperative emotional stress and pain, the consumption of anaesthetics and analgesics, and the duration of surgery and postoperative recovery [7,8]. Perioperative stress can cause nausea and fatigue, increase pain, and impair surgical wound healing and has significant effects on the immune system [7,9].

Hypnosis plays an important role in improving surgical wound healing and enhancing gastrointestinal recovery [7,9]. It is a cost-effective method and has no known adverse effects [10,11,12]. As part of a multimodal approach, it helps to reduce perioperative stress and pain, which in turn reduces medication consumption, recovery, and length of stay (LOS) [10].

PONV is one of the most common and undesirable side effects following general anaesthesia, with an overall incidence around 30% and can be as high as 80% in high-risk patients [13,14]. Prophylaxis for high-risk patients consists of a combination of two drugs with or without the inclusion of non-pharmacological methods [4,14].

The second most common adverse complication is postoperative delirium, which can be associated with a change in cognitive and mental status and can occur within minutes of surgery until discharge from the hospital [15,16]. After non-cardiac surgery, the incidence is between 15% and 54% [15]. It is even higher in critically ill patients [17]. It prolongs hospitalisation and increases cognitive decline and mortality, especially in elderly patients [15,16]. The factors most strongly associated with postoperative delirium include advanced age, pre-existing cognitive impairment (especially dementia), benzodiazepines (pre- and postoperative), perioperative anxiety and poorly managed postoperative pain [15,17]. The perioperative use of hypnosis and acupuncture alleviates pain and anxiety and consequently reduces the consmption of benzodiazepines and opioids, which in turn reduces the risk of postoperative delirium [1,7,9,11].

The multimodal approach to perioperative pain management or multimodal analgesia (MMA) is routinely used in anaesthesiology practice [18]. This involves combining several analgesic drug classes to achieve the desired analgesic effect while significantly reducing the incidence of drug-specific side effects [19]. It aims to reduce opioid consumption. The perioperative use of non-pharmacological methods, such as medical hypnosis and acupuncture, is also part of MMA [8]. Acupuncture reduces postoperative opioid consumption and pain [20,21]. On the other hand, hypnosis has a significant effect on the intensity of postoperative pain, but not on the reduction in analgesic consumption [8]. There is a strong correlation between preoperative anxiety and postoperative pain. Therefore, it is hypothesised that hypnosis and acupuncture together reduce the intensity of postoperative pain and opioid consumption not only directly but also indirectly by reducing preoperative anxiety [8,10,20]. To date, no study has investigated the simultaneous use of perioperative hypnosis and acupuncture in spinal surgery.

The aim of our study was to investigate the effects of acupuncture and medical hypnosis on the reduction in PONV and perioperative opioid consumption, length of stay (LOS), satisfaction, well-being, and complications.

## 2. Materials and Methods

The prospective, randomised study with three parallel groups was conducted from October 2021 to August 2022 at the University Medical Centre (UMC) Ljubljana. Surgical patients from the Clinical Department of Neurosurgery who underwent spinal surgery were included in the study. Exclusion criteria were minors, pregnant women, psychosis and unwillingness or inability to participate in the study.

The study was approved by the National Medical Ethics Committee of Slovenia (number NCT05068037, date 11 August 2022). It was registered by ClinicalTrials.gov (https://clinicaltrials.gov/study/NCT05068037 (accessed on 17 November 2025)). The article contains previously unpublished data from the study. All patients scheduled for spinal surgery were seen by a member of our team the day before surgery to obtain informed consent and answer any questions.

Randomisation and blinding: Prior to anaesthesia, patients were randomly assigned to one of three groups:

- HG (Hypno-acupuncture Group): Received hypnosis and acupuncture in addition to standard anaesthesia protocol, without ondansetron.
- HAG (Hypno-acupuncture + Antiemetic Group): Received hypnosis, acupuncture and ondansetron with standard anaesthesia protocol.
- CG (Control Group): Received standard premedication with midazolam and standard anaesthesia protocol with ondansetron, without hypnosis and acupuncture.

Randomisation was performed by a designated member of our study team. Two anesthesiologists, blinded to group allocation, were involved in the study. They performed the intraoperative protocol determined by the randomisation. The staff who performed the postoperative management and postoperative data collection did not know how the intraoperative management was performed or which patient group was involved. The data collected and the patient group were linked after data collection was completed.

### 2.1. Perioperative Management

Preoperative hypnosis protocol consisted of a single one-hour session, which included the standard anaesthetic assessment, a discussion about anaesthesia, an introduction to the study, an explanation of hypnosis as a relaxation technique before surgery, and signing of informed consent for both anaesthesia and study participation. Within this same session, a 30-min hypnosis was conducted.

The hypnotic trance was standardised and prepared by the author. It included induction, deepening, therapeutic suggestions with anchoring, awakening, and a post-hypnosis discussion focused on teaching self-hypnosis. The therapeutic imagery involved a “private sanctuary,” a place where positive healing energy, safety, freedom, and relaxation were reinforced.

Immediately before anaesthesia induction, the key suggestion was repeated—reviving the pleasant sensations of the private sanctuary—in order to reduce preoperative anxiety. Unlike in routine practice, where benzodiazepine premedication is often used, none was administered in this protocol.

In the CG, patients received standard premedication with midazolam on the day of surgery. Standard monitoring and an infusion line were placed. Induction and maintenance of anaesthesia were performed with TCI with propofol 4–6 µg/mL (Propoven, Fresenius Kabi AG, Bad Homburg, Germany) and remifentanil 4–6 µg/mL (Ultiva, GlaxoSmithKline, Parma, Italy). Rocuronium 0.6 mg/kg (Esmeron, MSD, New York, NY, USA) was used for muscle relaxation. The depth of anaesthesia was adjusted to ensure that the BIS value was 40–55.

In HG and HAG, acupuncture was performed bilaterally using sterile disposable needles (Seirin B-type, size 0.20 mm × 15 mm). The first point was PC6 (Neiguan), located on the inner forearm, approximately two cun proximal to the wrist crease, between the tendons of palmaris longus and flexor carpi radialis. Needles were inserted vertically or obliquely to a depth of 0.5–1 cun, depending on arm positioning.

The second point was LI4 (Hegu), located on the dorsum of the hand, between the first and second metacarpal bones, at the midpoint of the second metacarpal bone on the radial side. Needles were inserted vertically to a depth of 0.5–1 cun or obliquely to a depth of 1–1.5 cun.

Acupuncture was performed by licensed physicians who had completed formal training at the Slovenian School of Acupuncture, organised by the Slovenian Acupuncture Association under the auspices of the Slovenian Medical Association. The programme is accredited as part of the national continuing medical education system for physicians under the auspices of the Slovenian Medical Chamber.

After induction, all patients received 4 mg of dexamethasone. At the end of surgery, the patients in HAG and CG received the antiemetic ondansetron 4 mg. Piritramide 0.1 mg/kg (Dipidolor, Janssen-Cillag GmbH, Neuss, Germany) and metamizole 2.5 g (Analgin, Stada AG, Bad Vilbel, Germany) were administered at the beginning of wound closure. The reversal of neuromuscular blockade was performed with sugammadex 2–4 mg/kg (Bridion, MSD, Haarlem Waarderweg, The Netherlands) with regard to TOF measurement. The acupuncture needles were removed. After the operation, the patients were transferred to the postoperative care unit (PACU). Pain was assessed by an 11-point numerical rating scale (NRS) (0 meaning no pain, 10 meaning intolerable pain). Patients could receive an additional bolus of piritramide if the NRS was more than 3. For PONV, on request, patients in the HAG and CG could receive metoclopramide 10 mg and in HG, ondansetron 4 mg. The patients were then transferred to the neurosurgical intensive care unit (ICU).

Postoperatively, we recorded the following data: Length of stay in the hospital (LOS), consumption of analgesics and antiemetics, satisfaction and well-being, and complications.

Postoperative stress was assessed using patient-reported satisfaction (scale 1–5) and well-being (scale 0–5) scores as proxies for perceived stress. We monitored postoperative neurological status and noted non-neurological complications (PONV, pain, psychiatric events, infection, reoperation). Antiemetic consumption was our primary outcome; analgesic consumption, length of stay, patient satisfaction and well-being, and complications were secondary outcomes.

A standard postoperative analgesic protocol was used in all three groups, administering an intravenous infusion of piritramide as patient-controlled analgesia (PCA) in cervical spine surgery or tramadol with an intravenous flow regulator in lumbar spine surgery. Both in combination with metamizole (except in one patient who was known to be allergic to metamizole).

### 2.2. Statistical Analysis

Sample size was based on our previous observational data on antiemetic consumption across neurosurgery procedures (proportions 4.0%, 4.3%, 28.6%; Cohen’s w = 0.6). These findings informed the design of the current study, which aims to evaluate the efficacy of integrative interventions (hypnosis and acupuncture) in reducing PONV. A chi-square power analysis indicated that 20 participants per group yields an estimated power of approximately 80% power at α = 0.05.

**Figure 1:**
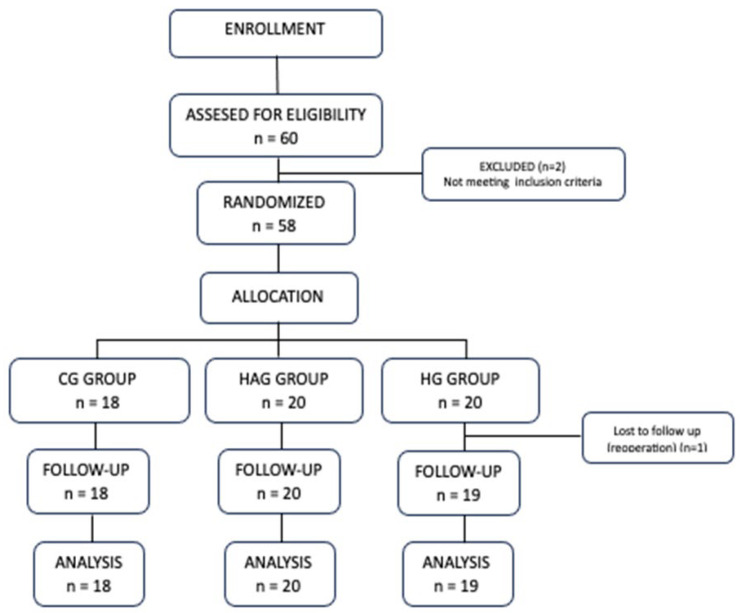
Flow chart of the study.

In addition to the a priori sample size calculation, a post hoc power analysis was conducted for the primary outcome (antiemetic consumption) using observed effect size and sample size. Two patients from the CG group and one from the HG group were excluded from further analysis (Figure 1).

Two-tailed *t*-test with unequal variances or chi-square test were used to test for differences in demographic data, postoperative analgesic and antiemetic consumption, patient well-being and satisfaction, length of stay and complications. The mean values of the continuous variables are presented, and the categorical data are summarised as counts. A *p*-value of less than 0.05 was considered statistically significant. The data were analysed using the SPSS 13.0 software package (IBM Corp., Armonk, NY, USA).

## 3. Results

In total, 60 patients aged 18–80 years, ASA (American Society of Anaesthesiologists) class 1–3, scheduled for cervical and lumbar spine surgery were included in the study, 20 in the CG group, 20 in the HAG group and 20 in the HG group (Figure 1). All surgeries were primary, and six of them were cervical spine surgeries; the others were lumbar spine surgeries. No significant differences (*p* > 0.05) were found between the groups in terms of their demographics, ASA class and type of surgery (Table 1).

There were no significant differences in the postoperative variables recorded in the first and 24 h after surgery. Only one patient in the HG required antiemetics after surgery; no additional antiemetics were given in the CG and HAG (*p* = 0.4).

Post hoc power analysis for antiemetic consumption indicated Cohen’s w = 0.189 and achieved power ≈ 23%, confirming underpowering due to low incidence.

Analgesic requirements were similar: additional opioids were administered in the first hour after surgery in most patients (17 in the CG and 16 in the HAG and HG; *p* = 0.4), but later in the ICU, only one patient in the HAG and two patients in the CG and HG required additional analgesics (*p* = 0.8) (Table 2). Overall satisfaction (9/9/0 vs. 10/6/4 vs. 9/7/3; *p* = 0.4) and well-being scores (10/8/0 vs. 13/5/2 vs. 13/5/1; *p* = 0.5) were high across all groups. Two patients in the CG had mild psychiatric complications after surgery. The length of hospital stay was comparable (3 days CG vs. 4 days HAG and HG (*p* = 0.7) (Table 3).

There were no significant differences in the postoperative variables recorded in the first and 24 h after surgery. Only one patient in the HG required antiemetics after surgery; no additional antiemetics were given in the CG and HAG (*p* = 0.4).

Although not statistically significant, the need for rescue antiemetics was minimal in all groups, indicating similar effectiveness between the non-pharmacological and pharmacological approaches.

## 4. Discussion

Numerous studies have shown that hypnosis or self-hypnosis before surgery effectively reduces emotional stress, the fear response and the memory of unpleasant experiences [10,11,22,23]. In our study, patients in the HG and HAG groups did not receive preoperative anxiolytic benzodiazepines but were hypnotised preoperatively. We found no differences between the groups in the assessment of satisfaction and well-being before and after surgery, which were rated highly. This suggests that preoperative hypnosis was as effective as sedation with midazolam in reducing anxiety in our patients undergoing spinal surgery.

The use of preoperative hypnosis is associated with less postoperative nausea and vomiting and a shorter recovery time after surgery [11,24,25]. However, more frequent and longer hypnosis sessions did not offer significant advantages over single, shorter hypnosis sessions [7].

Hypnosis affects the neural connections in the cortical and subcortical areas involved in pain signalling and pain perception, which may lead to pain relief [26]. Several studies have shown that the use of hypnosis before surgery effectively reduces pain, decreases the release of stress hormones and reduces the use of analgesics and their side effects [10,11,23]. The effect of medical hypnosis on reduced opioid consumption was demonstrated in patients who had received opioid therapy prior to surgery, but not in opioid-naive patients [27].

We found no significant differences in the use of antiemetics and analgesics. There was no increased incidence of PONV in the HG group, in which patients received only one antiemetic, but preoperative hypnosis and intraoperative acupuncture. In the other two groups (HAG and CG), patients received double antiemetic protection. However, during the study, only one patient required additional antiemetic therapy. The lack of statistically significant differences in antiemetic use may be attributed to the study’s limited sample size and low event rate. Post hoc power analysis confirmed insufficient power to detect statistically significant differences, underscoring the need for larger studies, and findings should be interpreted with caution.

The literature suggests that the intraoperative use of acupuncture is effective in reducing PONV. Acupuncture is effective in the prevention and treatment of PONV in both children and adults [2,5,28,29,30]. The acupuncture point Neiguan (PC 6) is supported for PONV prevention [29]. Our results align with prior evidence, but timing after induction may have a limited effect.

Stimulation of this point can reduce the intraoperative use of opioid analgesics in laparoscopic procedures where the prevalence of PONV is high [31]. PC 6 stimulation can serve as a non-pharmacological alternative to antiemetics such as ondansetron, droperidol and metoclopramide [32,33,34]. The combination of PC 6 with other acupuncture points such as Hegu (LI 4), Zusanli (ST 36) and auricular points is even more effective in the prevention and treatment of PONV [5,34]. Likely mechanisms for reducing the incidence of PONV include changes in serotonin transmission and the release of beta-endorphins [34]. The favourable antiemetic effect of acupuncture could be exploited in ambulatory surgery, in the elderly and in patients at risk with “triple low” (low MAC, MAP and BIS) [35].

As part of multimodal analgesia and to reduce opioid consumption, it is recommended to administer ketamine or dexmedetomidine intraoperatively and to infiltrate the surgical field with a local anaesthetic [36]. Acupuncture works in the same way and can be an integral part of multimodal analgesia. To the extent that intraoperative acupuncture prevents activation of pain pathways and produces analgesia, it can also reduce postoperative pain and the need for postoperative opioids [37,38,39]. Intraoperatively, it reduces the required dose of opioids and provides more comfortable postoperative conditions than anaesthesia alone [36,40]. Acupuncture-induced postoperative analgesia may last 6–12 h, but no longer than 24 h. The inability of acupuncture to reduce morphine consumption for more than 24 h may be explained by the fact that the analgesic effect of a single electroacupuncture session lasts only two to three hours [41].

However, in our study, the intraoperative use of acupuncture had no effect on the use of analgesics in the early postoperative period. We were also unable to demonstrate a reduction in perioperative opioid consumption. The reasons for this could be: Performance of acupuncture after induction, selection of acupuncture points, hyperalgesia after remifentanil, and excessive routine administration of opioids in the PACU.

In the study by Gupta and colleagues, acupuncture performed 15 min before induction of anaesthesia did not result in a reduction in the use of analgesics [42]. In children undergoing tonsillectomy, perioperative acupuncture reduced pain, decreased the use of analgesics and improved patient satisfaction [43]. The results of studies on the effects of acupuncture on perioperative pain are very contradictory [29,30,42,44,45]. The timing of acupuncture may also influence the results. Some researchers have recommended that acupuncture be performed prior to induction of anaesthesia and/or in the early postoperative period [21,46,47].

Both manual and electrical stimulation techniques can produce the de qi sensation. In fact, studies have shown that electrical stimulation provides better pain relief [48].

It is likely that in our patients, stimulation of acupuncture points after surgery is necessary to achieve the desired analgesic effect. Furthermore, electroacupuncture could enhance this effect.

Additionally, remifentanil-induced hyperalgesia may have masked the analgesic effects of hypnosis and acupuncture. This phenomenon, linked to NMDA receptor activation and central sensitisation, is well-documented in the literature. A recent meta-analysis confirmed that higher intraoperative doses of remifentanil correlate with increased postoperative pain [49].

These confounding factors should be considered when interpreting our findings and highlight the need for future studies with optimised analgesic protocols and better control of opioid-related hyperalgesia.

The prior study was designed to establish a standard antiemetic protocol that included two pharmacologic agents—dexamethasone and ondansetron—to minimise the risk of postoperative nausea and vomiting (PONV), particularly in neurosurgical procedures, which are considered high-risk for PONV. Based on those results, our current study retained dexamethasone as a baseline intervention and explored the addition of hypnosis and acupuncture as integrative strategies.

Despite the statistical limitations, the clinical outcome is highly encouraging. Neurosurgical procedures are known to carry a high risk for PONV, yet our findings showed near-complete absence of PONV across all groups—including the group without ondansetron—suggesting that acupuncture combined with dexamethasone may be as effective as standard pharmacologic protocols.

Another fact is that after lumbar spine surgery, we use tramadol for postoperative analgesia. Tramadol and ondansetron are known to interact pharmacodynamically, potentially reducing tramadol’s analgesic efficacy due to serotonergic pathway interference. The ability to avoid ondansetron in the integrative groups not only maintained PONV control but also preserved tramadol’s analgesic efficacy, supporting a safer and more streamlined postoperative protocol.

Tramadol also enables a strong opioid sparing effect and aligns with current goals in perioperative medicine to minimise opioid exposure. We believe this outcome strengthens the clinical relevance of our findings and supports protocol refinement.

Use of a weak opioid in combination with acupuncture and hypnosis contributes to a strong opioid sparing effect.

This supports a shift toward more personalised, multimodal, integrative approaches like hypnosis and acupuncture in perioperative care, reducing pharmacologic burden while maintaining clinical efficacy.

Although the study was underpowered to detect statistically significant differences in PONV, the low incidence observed across all groups suggests that non-pharmacological interventions may offer comparable clinical benefit. These findings warrant further investigation in larger, multicenter trials. This exploratory study highlights the feasibility and potential value of integrating hypnosis and acupuncture into multimodal perioperative care. Future research with larger sample sizes is needed to confirm these preliminary observations.

## 5. Conclusions

The standard anaesthetic protocol effectively managed perioperative stress and pain. Within this exploratory trial, hypnosis appeared comparable to benzodiazepines for anxiety reduction, and acupuncture did not increase PONV despite reduced pharmacological prophylaxis. No significant differences were detected in opioid or antiemetic use. It is difficult to compare our results with other studies because there are very few large multicenter studies on this topic. In addition, we would need a larger number of patients to draw relevant conclusions.

These results highlight the need for larger, multicenter trials to validate the clinical relevance of non-pharmacological interventions in multimodal care.

Limitations: This study has several limitations. First, the incidence of postoperative nausea and vomiting (PONV) was extremely low (only one patient required rescue antiemetics), which precluded meaningful statistical comparison for this outcome. Post hoc power analysis confirmed that the study was underpowered for detecting differences in antiemetic use (observed effect size Cohen’s w = 0.189; achieved power ≈ 23%).

Second, the absence of single-modality groups (hypnosis-only or acupuncture-only) limits the ability to isolate the individual contributions of each intervention.

Third, acupuncture was performed after induction of anaesthesia, which may have reduced its psychophysiological impact compared to preoperative application.

Finally, routine perioperative opioid administration and remifentanil use could have masked the potential analgesic benefits of the non-pharmacological interventions. These factors should be considered when interpreting the findings and designing future studies.

## Figures and Tables

**Table 1:** Baseline demographics and type of surgery.

GROUP	CG (n = 18)	HAG (n = 20)	HG (n = 19)	*p*
**Age (years)**	60 ±12	60 ± 10	61 ± 13	0.9
**Gender (male/female)**	10/8	14/6	10/9	0.5
**Weight (kg)**	89 ± 15	87 ± 12	79 ± 13	0.1
**Height (cm)**	173 ± 8	175 ± 9	170 ± 11	0.2
**ASA (1/2/3)**	3/13/2	1/17/2	3/12/4	0.6
**Type of surgery (CS/LS)**	0/18	4/16	2/17	0.1

The results are expressed as mean ± SD or number of patients. The differences between groups were not significant (*p* > 0.05). Abbreviations: ASA, American Society of Anaesthesiologists; CS, cervical spine; LS, lumbar spine.

**Table 2:** Additional analgesics and antiemetics in PACU and ICU.

GROUP	CG (n = 18)	HAG (n = 20)	HG (n = 19)	*p*
**Additional analgesic PACU (yes/no)**	17/1	16/4	16/3	0.4
**Additional analgesic ICU (yes/no)**	2/16	1/19	2/17	0.8
**Additional antiemetic PACU (yes/no)**	0/18	0/20	1/18	0.4
**Additional antiemetic ICU (yes/no)**	0/18	0/20	1/18	0.4

The results are expressed as mean ± SD or number of patients. The differences between groups were not significant (*p* > 0.05). Abbreviations: ICU—intensive care unit; PACU—postoperative care unit.

**Table 3:** Well-being, satisfaction of the patients, length of stay and complications.

GROUP	CG (n = 18)	HAG (n = 20)	HG (n = 19)	*p*
**Well-being (perfect/very good/good)**	9/9/0	10/6/4	9/7/3	0.4
**Satisfaction (perfect/very good/good)**	10/8/0	13/5/2	13/5/1	0.5
**LOS (days)**	3 ± 1	4 ± 2	4 ± 2	0.7
**Complications (no/yes)**	16/2	20/0	19/0	0.1

The results are expressed as mean ± SD or number of patients. The differences between groups were not significant (*p* > 0.05). Abbreviations: LOS—length of stay.

## Data Availability

The data used in this study are available from the corresponding author upon reasonable request. The data are not publicly available due to the inclusion of confidential patient information in paper-based records and institutional restrictions.

## Abbreviations

The following abbreviations are used in this manuscript:

ASA	American Society of Anaesthesiologists
CS	Cervical Spine
LS	Lumbar Spine
PACU	Postoperative Care Unit
LOS	Length of the Day
PONV	Postoperative Nausea and Vomiting
HG	Hypno-acupuncture Group
HAG	Hypno-acupuncture Group with Antiemetic
CG	Control Group
UMC	University Medical Centre
ICU	Intensive Care Unit
LOS	Length of Stay
MMA	Multimodal Analgesia
TCI	Target-Controlled Infusion
TOF	Train-of-Four
NRS	Numerical Rating Scale
PCA	Patient-Controlled Analgesia
